# Modern Innovative Solutions to Improve Outcomes in Severe Asthma: Protocol for a Mixed Methods Observational Comparison of Clinical Outcomes in MISSION Versus Current Care Delivery

**DOI:** 10.2196/resprot.9585

**Published:** 2019-10-10

**Authors:** Claire Roberts, Eleanor Lanning, Carole Fogg, Paul Bassett, Alison Hughes, Anoop J Chauhan

**Affiliations:** 1 Portsmouth Hospitals NHS Trust Portsmouth United Kingdom; 2 University of Portsmouth Portsmouth United Kingdom; 3 Stats Consultancy Berkshire United Kingdom

**Keywords:** asthma, diagnosis, community health services, drug therapy, epidemiology, asthma treatment

## Abstract

**Background:**

Asthma that is poorly controlled and undertreated can progress to more severe disease that is associated with high levels of unscheduled care that requires high-cost therapy, leading to a significant health economic burden. The identification and appropriate referral to a specialist asthma service is also often delayed by several months or years because of poor recognition and understanding of symptom severity. Current severe asthma services may take several months to provide a comprehensive multidisciplinary assessment, often necessitating multiple hospital visits and costing up to £5000 per patient.

**Objective:**

This study aims to evaluate whether a new service model could identify poorly controlled and potentially severe asthma much earlier in the patient pathway, and then compare clinical outcomes between this new care model with standard care.

**Methods:**

Modern Innovative Solutions to Improve Outcomes in (MISSION) Severe Asthma is a novel service model developed by asthma specialists from Portsmouth and Southampton severe asthma services. MISSION Severe Asthma identified patients with poorly controlled disease from general practice databases who had not been under secondary outpatient care in the last 12 months or who were not known to secondary care. In 1- or 2-stop assessments, a thorough review of diagnosis, disease phenotype, and control is undertaken, and clinical outcomes collected at baseline.

**Results:**

A variety of clinical outcomes will be collected to assess the service model. The results will be reported in February 2020.

**Conclusions:**

This protocol outlines a mixed methods study to assess the impact on disease control, unscheduled health care usage, and quality of life in patients seen in the MISSION clinic compared with a closely matched cohort who declined to attend.

**International Registered Report Identifier (IRRID):**

DERR1-10.2196/9585

## Introduction

### Background and Rationale

#### The Burden of Disease

Asthma affects 5.4 million patients in the United Kingdom with the majority of costs (nearly 80%) relating to treating poorly controlled asthma, amounting to over £1 billion per annum as a direct cost and an indirect cost to society (time off work and lost productivity) of £6 billion. In the past year, the Wessex Region has seen over 1800 emergency adult hospital admissions because of asthma (over 300 more than expected based on the national average), costing over £2.1 million to the local health authority.

Asthma UK highlights that over half of the people with asthma suffer debilitating symptoms despite being prescribed treatment [1]. Poorly controlled disease leads to exacerbations necessitating unscheduled care and high-cost medications while impairing a patient’s quality of life and increasing the risk of premature death. In 2009, there were 1131 deaths because of asthma in the United Kingdom, triggering a Department of Health–commissioned National Audit of Asthma Deaths [2]. Reviews of asthma deaths have confirmed that those with the most severe, frequently exacerbating disease are at greatest risk of death. The Outcome Strategy for Chronic Obstructive Pulmonary Disease and Asthma recognized this huge burden on both patients and the National Health Service (NHS) and outlined the political commitment to improve asthma control and reduce asthma-related emergency health care needs and deaths [3]. Asthma UK also highlighted the need to improve the quality of life of people with asthma by improving access to services, reducing inequalities in care, and ensuring a high standard of care. It is accepted that the proactive clinical review of people with asthma improves clinical outcomes and the majority with poorly controlled asthma can achieve good control. The recent publication “NHS five-year forward view” identifies the traditional separation of primary and secondary care being a barrier to the integrated care that patients with chronic disease require [4].

#### The Unmet Need

The Specialised Services Pathway for Severe Asthma recognizes the burden of uncontrolled disease ordinarily amenable to antiinflammatory medications and the progression to longer term “severe” disease, necessitating high-cost therapies (e.g. bronchial thermoplasty) [5]. The identification and appropriate referral to a specialist asthma service is often delayed by several months or years because of poor recognition and understanding of symptom severity.

Current severe asthma services may take several months to provide a comprehensive multidisciplinary assessment often necessitating multiple hospital visits and costing up to £5000 per patient. The report “Fighting for Breath” highlighted these issues with service delivery recommending that referral pathways to severe asthma services should be improved [6].

#### Description of Clinical Intervention and Treatment

Modern Innovative Solutions to Improve Outcomes in (MISSION) Severe Asthma is a novel service model pilot that took place in June and July 2014. MISSION was developed by asthma specialists from Portsmouth and Southampton asthma services.

The MISSION concept involved a “carousel clinic” which brings together all the assessments and investigations a patient requires into one clinic—the patient moved around each assessment or “investigation station” in a serial fashion. This allowed the research team to efficiently gather all the data required for both staff and the patient. This severe asthma patient journey was mapped and opportunities for improvement were identified such as earlier diagnosis by active case finding of at-risk patients, followed by a swift multidisciplinary assessment which meant patients could be seen sooner and assessed more effectively. Patients attending standard outpatient services undergo basic lung function, chest x-ray, and medical review. They are then referred for other tests or specialist opinions as required at different time-points

MISSION Severe Asthma identified patients with poorly controlled disease from general practice (GP) databases who had not been under secondary outpatient care in the last 12 months or who were not known to secondary care. The patients were then invited to Rapid Access Asthma Clinics (RAACs) for assessment at their GP surgery on a Saturday, at a time convenient for them. The clinics were held on Saturdays at a time better suited to the needs of asthma patients, many of whom are working or studying full-time.

**Figure 1:**
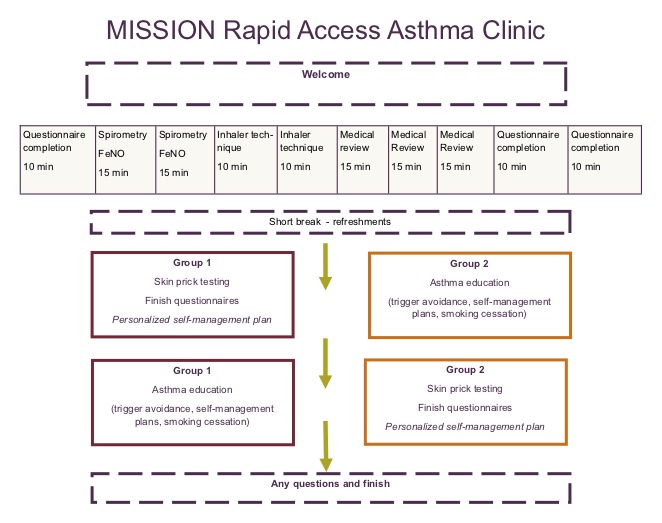
MISSION Severe Asthma Rapid Access Asthma Clinic flow chart. MISSION: Modern Innovative Solutions to Improve Outcomes.

The RAAC delivered a comprehensive asthma review including assessments of allergic status, airway inflammation, education, self-management planning, and smoking cessation leading to a written personalized 1-year asthma management plan for their GP providing clear treatment recommendations for those with uncontrolled but treatable asthma (see Figure 1). Treatment recommendations were in line with current asthma guidelines.

At the end of the RAAC, a multidisciplinary team (MDT) meeting was held and treatment plans were discussed. The possible outcomes for patients included increasing or decreasing medication, referral for research study, referral to other medical teams, or a need for further assessment as the patient has potentially severe and uncontrolled asthma. Patients with potentially severe and uncontrolled asthma were invited for a more detailed multidisciplinary specialist assessment at a Severe Asthma Assessment Clinic (SAAC) again on a Saturday, held at Queen Alexandra Hospital.

**Figure 2:**
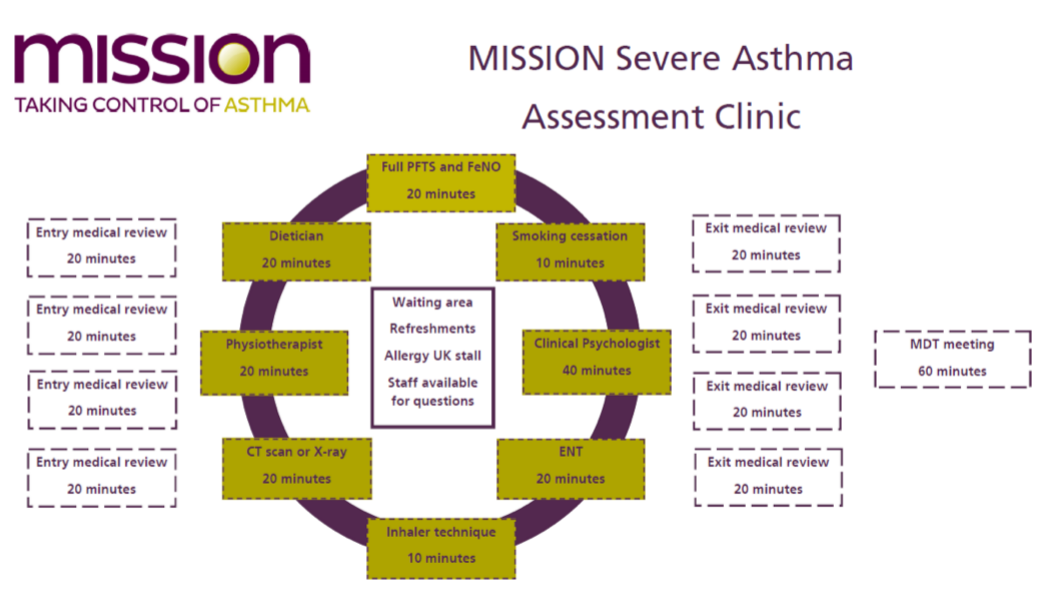
MISSION Severe Asthma Rapid Access Asthma Clinic flow chart. CT: computed tomography; ENT: ear, nose, and throat; FeNO: fractional exhaled nitric oxide; MDT: multidisciplinary team; MISSION: Modern Innovative Solutions to Improve Outcomes; PFT: pulmonary function test.

MISSION SAAC included multidisciplinary assessment respiratory specialists, dieticians, physiotherapists, ear, nose, and throat (ENT) consultants, physiologists, psychologists, and pharmacists. Computed tomography (CT) scanning of the chest and sinus as well as flexible nasendoscopy to assess for sinus change and nasal polyps was available on that day. Patients saw the appropriate specialists based on the clinical history from the RAAC and a medical assessment on arrival to the SAAC. This ensured full lung function, inhaler technique, and medical exam for all patients, and physiotherapy, dietician, ENT, CT chest or sinus were performed where needed (see Figure 2). Representatives from Allergy UK also attended the clinic and provided advice and support to patients who were interested.

At the end of the clinic, a full MDT meeting was held to discuss each case. Patients and their GP received a detailed letter with all the test results and treatment changes as well as plans for follow-up. Follow-up referrals were made to specialists as needed, and treatment changes were made on the day of the clinic visit with support from the pharmacy for new treatment prescriptions.

In summary, the difference of the MISSION clinic from standard care was in 2 areas: First the active case finding of patients who are not already known to secondary care but are potentially uncontrolled. The second is the model of the clinic. Patients seen at the RAAC only will undergo more extensive testing and review of their asthma than primary care can provide and will have their comorbidities identified, asthma phenotyped, and treatment changes made appropriately.

#### Rationale for Study and Potential Impact

MISSION is a new and novel way of delivering highly specialized asthma care and has the potential to change the way asthma care is delivered across the United Kingdom as well as services for other long-term health conditions. The MISSION model is the first model of this type, and this study aimed to evaluate its success and compare the MISSION service with current care delivery. This will be done in several different ways. The study is a mixed methods evaluation of the new service comparing outcomes before and after the MISSION clinic using retrospective data analysis and prospective qualitative interviews. A control arm of patients not exposed to, but eligible for, the new MISSION clinic will also be included.

### Objectives

Using quantitative and qualitative methods, our objective is to explore the impact of the MISSION service on clinical outcomes and patient experience. Using quantitative methods, we will (1) retrospectively analyze data collected as part of routine clinical care from all patients attending MISSION e.g. asthma control, medication usage and technique, exacerbations, comorbidities, allergies, and investigations (blood tests, radiological imaging, and nasendoscopy) and (2) retrospectively compare the assessment of comorbidities, investigations, and treatments between MISSION SAAC patients and standard outpatient care.

#### Primary Objectives

The primary objectives of this study are as follows:

1. To assess whether asthma control (assessed by exacerbation history, Asthma Control Questionnaire (ACQ), health care utilization, and short-acting beta-agonist (SABA) usage) is improved in MISSION SAAC patients compared with patients undergoing standard outpatient secondary asthma care at 6 months.
2. To assess whether asthma control (assessed by exacerbation history, health care utilization, and SABA usage) is improved in MISSION RAAC patients compared with those who were eligible for MISSION but did not attend.
3. To assess whether asthma control (assessed by exacerbation history, health care utilization, and SABA usage) is improved after attending the MISSION RAAC and SAAC.

#### Secondary Objectives

1. To conduct a prospective qualitative study exploring patients’ experience, understanding and perceptions of their asthma as well as views on MISSION.
2. To explore the acceptability of MISSION as a service model for patients, staff, and the NHS.
3. To retrospectively compare a clinical case finding approach in primary care with a computer-assisted interrogation on primary care records.
4. To assess the health economic impact of the MISSION service.
5. To phenotype patients with uncontrolled or severe asthma in primary care and gain more information about the asthma population in Wessex.
6. To assess whether asthma comorbidities are assessed in standard outpatient care.
7. To assess waiting times and times to specialist referral in MISSION and standard outpatient care.

## Methods

### Study Design

A mixed methods observational study evaluating the new service, comparing outcomes before and after the intervention, and including a control arm of patients not exposed to, but eligible for, the new intervention is included. This will include analyses of the MISSION participants (attending both the RAAC and SAAC clinics), patients from the existing asthma service (from outpatient severe asthma clinics), and data from patients on GP records who were eligible to attend MISSION but did not (primary care patients). Finally, qualitative methods will be used to explore participant and health care professionals’ views on MISSION. Multimedia Appendix 1 summarizes the participants journey and the different populations under study.

The MISSION Severe Asthma project has not been designed as a randomized controlled trial (RCT) but designed to evaluate a novel service model (the intervention) in participants with uncontrolled and potentially severe asthma, to assess whether it is deliverable and acceptable by participants and health care professionals, and to explore the barriers that would need to be addressed before a larger RCT.

A patient advisor reviewed all patient-facing documentation for the study and will give advice and feedback on the structure of the qualitative interviews.

### Study Participants

Participants with uncontrolled or potentially severe asthma were identified from GP records by the MISSION team and new referrals to an asthma specialist clinic at Queen Alexandra Hospital between May and August 2014.

Study participants for qualitative interview will be recruited from patients who attended MISSION SAAC days and staff who attended MISSION RAAC or SAAC days. Qualitative one-to-one interviews will be held over the telephone.

Data for quantitative analysis will be collected from records made by the clinical team during the MISSION RAACs and SAACs and asthma outpatient clinics. GP records will be entered as usual by the GP. No extra data will be collected.

### Eligibility Criteria

The participant must meet all of the following criteria to be considered eligible for the study:

- Male or female, aged 18 years or older.
- Is in one of the following population groups:
  - Attended the MISSION RAAC or
  - Attended the MISSION SAAC or
  - Identified as uncontrolled asthma by record searches and invited to the MISSION RAAC but did not attend “primary care patients” or
  - Has been referred to the specialist asthma clinic at Queen Alexandra Hospital “outpatient severe asthma patients” or
  - Attended the MISSION RAAC or SAAC as a health care professional
- Participant is willing and able to give informed consent for participation in the study.

### Primary and Secondary Endpoints and Outcome Measures for Quantitative Study

#### Primary Outcome Measure for All Patients

The primary endpoint is asthma control as measured by exacerbation frequency (defined as deterioration in symptoms requiring ≥30 mg prednisolone or equivalent for ≥3 days).

#### Secondary Outcome Measures for Modern Innovative Solutions to Improve Outcomes in Severe Asthma Assessment Clinic Patients and Outpatient Severe Asthma Clinic Patients

1. SABA use measured by the number of inhalers prescribed in 6 months pre- and post-MISSION or outpatient clinic.
2. Exacerbation frequency (defined as deterioration in symptoms requiring ≥30 mg prednisolone or equivalent for ≥3 days) in 6 months pre- and post-MISSION or outpatient clinic.
3. Health care usage costs for asthma and number of contacts (GP visits, emergency department [ED] or out-of-hour [OOH] attendances, hospital admissions, and emergency GP visits) over 6 months pre- and post-MISSION or outpatient clinic.
4. Assessment of comorbidity (rhinosinusitis, anxiety and depression, dysfunctional breathing, gastro esophageal reflux, and obstructive sleep apnea) and method of assessment.
5. Assessment of inhaler technique and recommendations for inhaler devices.
6. Smoking cessation advice.
7. Investigations performed during 6 months in secondary care, for example, full lung function, sputum induction, and performing a high-resolution CT scan of the chest.
8. Time from GP referral to first clinic visit in secondary care.
9. Time between first and second visit in secondary care.
10. Time to appointment with other specialists for asthma-related comorbidity where indicated, for example, dietician, ENT, physiotherapist, psychologist, and CT imaging.
11. Assessment of eosinophilic airways inflammation through measurement of fractional exhaled nitric oxide (FeNO).
12. The frequency of nonattendance at clinic.

#### Secondary Outcome Measures for Modern Innovative Solutions to Improve Outcomes in Rapid Access Asthma Clinic Patients

1. SABA use measured by the number of inhalers prescribed in 6 months pre- and post-MISSION clinic.
2. Exacerbation frequency (defined as deterioration in symptoms requiring ≥30 mg prednisolone or equivalent for ≥3 days) during the 6 months pre- and post-MISSION.
3. Frequency and severity of comorbidities.
4. Frequency and type of allergy.
5. Measurement of exhaled nitric oxide.
6. Measurement and variation of lung function.
7. Frequency and type of additional asthma control medication.
8. Disease control and quality of life as assessed by ACQ and Asthma Quality of Life Questionnaire (AQLQ).
9. Number of patients having measurement of forced expiratory volume in 1 second (FEV1)÷forced vital capacity (FVC) as a proxy for asthma control and severity.

#### Secondary Outcome Measures for Modern Innovative Solutions to Improve Outcomes in Rapid Access Asthma Clinic Patients and Primary Care Patients

1. Prescription of SABAs during last 6 months at baseline and 6 months.
2. Exacerbation rates (defined as deterioration in symptoms requiring ≥30 mg prednisolone or equivalent for ≥3 days) during the last 6 months at baseline and 6 months.
3. ED attendances, OOH contacts, and hospital admissions during the last 6 months at baseline and 6 months.
4. Inhaled steroid doses and usage.
5. Number of patients having measurement of FEV1÷FVC as a proxy for asthma control and severity.
6. The sensitivity and specificity of the PRIMIS (Primary Care Information Services, University of Nottingham) Asthma Audit Tool in identifying the patients compared with gold standard specialist assessment and interrogation of primary care records.

### Screening and Enrollment

#### Quantitative

All patients from the RAACs and all primary care patients will be approached for consent to use their anonymized data from the MISSION clinics or from GP records for research purposes. No extra data for research purposes will be collected for the quantitative analysis from patients at MISSION assessments, asthma outpatient clinics, or GPs.

#### Qualitative

##### Patients

All patients who attended MISSION SAACs will be approached to take part in qualitative one-to-one telephone interviews.

##### Health Care Professionals

Health care professionals who worked at the MISSION clinics from both primary and secondary care will be approached for qualitative interview. All health care professionals will be approached with an estimated 10 to be interviewed from primary care and 10 from secondary care. They will be contacted by email or telephone and, if interested, they will be sent a written information sheet by email or post. They will also be given contact details if they have any further questions regarding what is required to take part. If willing to take part, they will be sent a consent form to fill in and return to the study team.

### Recruitment

#### Modern Innovative Solutions to Improve Outcomes in Rapid Access Asthma Clinic and Severe Asthma Assessment Clinic Patients

All patients who attended the MISSION RAACs (quantitative) and SAACs (quantitative and qualitative) will be contacted by telephone to discuss the study and then, if they are interested, will be sent a patient information sheet (PIS) and consent form with a covering letter.

If the patient is unable to be contacted by telephone, they will be sent a covering letter from the MISSION team inviting them to take part in the study. They will be provided with a number to contact if they have any questions. They will be given the opportunity to read the PIS, and if they agree to take part in the study, they can return the consent form by post in a stamped addressed envelope.

#### Primary Care Patients

Patients in primary care will be contacted as described above.

#### Outpatient Severe Asthma Patients

Patients who attended outpatient clinics as new referrals in June and July will be approached by telephone after being identified from the clinic attendance list. If the patient is willing, they will be sent a PIS and consent form to send back to the study team in a stamped addressed envelope.

#### Health Care Professionals

Health care professionals who attended the MISSION RAACs or SAACs will be contacted by email or telephone to explain the study and then sent a PIS by email or post if they are interested in taking part. They will be given time to read the PIS, and if they wish to take part, they will be asked to return a consent form in a stamped addressed envelope.

### Quantitative Data Collection

Retrospective notes review at 6 months after the MISSION clinics will be performed to collect the following data. Data collected by study group are detailed in Table 1. Where information is not written in medical notes or clinic letters, it will be marked as not assessed.

**Table 1:** Schedule of procedures at the Severe Asthma Assessment and Rapid Access Asthma Clinics.

Study group	MISSION^a^ Severe Asthma Assessment Clinics and outpatient severe asthma	MISSION Rapid Access Asthma Clinics and primary care patients
Full medical and asthma history	✓^b^	✓
Lung function testing	✓	✓
Inflammometry	✓	✓
Medication history	✓	✓
Health care usage (general practitioner attendances, emergency department attendances, and admissions)	✓	✓
Allergy status	✓	✓
Smoking status and history	✓	✓
Inhaler technique before and after review and documentation of improvements made	✓	✓
Disease-specific scores: Asthma Control Questionnaire and Asthma Quality of Life Questionnaire	✓	✓
Comorbidity screening: Epworth Sleepiness Scale, Sino Nasal Outcome Test -22, Hospital Anxiety and Depression Scale, Gastroesophageal Reflux Disease Questionnaire, and Nijmegen	✓	✓
Reviews by wider asthma multidisciplinary team, including time from referral to review	✓	X^c^

^a^MISSION: Modern Innovative Solutions to Improve Outcomes.

^b^✓: assessment performed.

^c^X: assessment not performed.

#### Modern Innovative Solutions to Improve Outcomes in Severe Asthma Assessment Clinic Patients and Outpatient Severe Asthma Patients

Retrospective notes review at 6 months after the MISSION clinics will be performed to collect the following data for MISSION SAACs and asthma outpatients. The clinical record form used for the MISSION SAACs is shown in Multimedia Appendix 2.

Where information is not written in medical notes or clinic letters, it will be marked as not assessed. Information will include the following:

- Medical history including asthma history, asthma triggers, allergy history, past medical history, family history, and occupation.
- Lung function including peak flow, FEV1, FVC, transfer factor of the lung for carbon monoxide, CO transfer coefficient, and FeNO.
- Medication history including asthma and non-asthma medication.
- Exacerbation history including number of steroid courses, hospital admissions, and intensive treatment unit admissions.
- Health care usage including nonroutine GP attendances, OOH contacts, and ED attendances in 6 months pre- and post-MISSION SAAC or outpatient clinic.
- Allergy testing results and whether done.
- Smoking status, and if current smoker, whether any smoking cessation advice or referral was done.
- Inhaler technique—whether checked, any improvements made, and recommendations for inhaler devices.
- Investigations performed and time in days after first visit.
- ACQ score.
- AQLQ score.
- Sino nasal outcome test-22 (measure of sino nasal symptoms) score.
- Epworth sleepiness score (measure of somnolence, which can be caused by obstructive sleep apnea).
- Hospital anxiety and depression scale score.
- Nijmegen (assessment of hyperventilation syndrome) score.
- Gastroesophageal Reflux Disease Questionnaire (assessment of gastroesophageal reflux) score.
- Time of GP referral, first clinic visit, and second clinic visit.
- Time of referral and appointment with other specialities, for example, ENT, dietician, and physiotherapy.

#### Modern Innovative Solutions to Improve Outcomes in Rapid Access Asthma Clinics

- Lung function including FEV1, FVC, peak flow, and FeNO.
- ACQ and AQLQ at baseline, 3 months, and 6 months in MISSION RAAC patients.
- Medication history for the last 12 months.
- Exacerbation history for the last 12 months.
- OOH, hospital, and emergency department attendances

### Qualitative Data Collection

#### Modern Innovative Solutions to Improve Outcomes Severe Asthma Assessment Clinic Patients

The target population for the qualitative telephone interviews is all of the 20 patients who attended the MISSION SAACs. All patients in this group will be approached for an interview. Participants from MISSION SAACs who are taking part in qualitative interviews will have 1 telephone interview as part of this evaluation research study.

Interviews will be arranged at a mutual date and time of convenience. The researcher will make the telephone calls to minimize costs to the participants and facilitate audio recordings. Interviews will be held in a semistructured manner using a topic guide with relevant prompts. This will allow patients to focus discussion on the issues important to them and hear their experiences and views in their own words with free discussion guided by the patient.

The areas explored will include the patients’ perceptions, understanding, fears, and concerns about their asthma as well as their views on MISSION—problems, barriers, and positive experiences. The interview will explore asthma and asthma service delivery.

The topic guide will be developed in conjunction with the payment protection insurance advisor.

#### Health Care Professionals

Telephone interviews will also be held with medical staff from both primary and secondary care who worked at the clinics. The anticipated number of participants will be 10 from primary care and 10 from secondary care. All staff who attended the MISSION clinics will be approached for consent. The interviews will explore the health care professionals’ thoughts on MISSION and its strengths and weaknesses as well as any suggestions for improvement. The interviews will also explore the health care professionals’ understanding and training in severe asthma and any areas where they would wish to see improvement.

#### Interview Structure

The semistructured interviews will be conducted by telephone and audio-recorded with notes taken. Telephone interviews have been chosen as participants live over a wide geographical area, and it will allow flexibility with interview times to accommodate participants’ work and other commitments.

The interviews will be conducted by one of the health care professionals involved in the clinic. By having an existing relationship with the study participants and by the interviewer being a health care professional, the participant may be more willing to share experiences and personal views [7,8]. The researcher will be aware of the potential conflict with the professional role and the role as interviewer and will ensure open questions are asked [9]. The skills required during a clinical consultation have considerable overlap with the skills in qualitative interview [7,9]. It is important that the relationship between the interviewer and interviewee is clear when planning the qualitative interview [10] as it is recognized that this can be a factor in the interview.

At the beginning of the interview, patient participants will be reminded that the interview topic guide can be deviated, that they are free to suggest topic areas relevant to their experience and tell their experiences in their own words, and that there are no right or wrong answers but to share with the interviewer their thoughts, both the positives and negatives about MISSION, living with asthma, and asthma care.

There is also a concern that the patient participants may raise health questions or ask for health care advice. This will be addressed by explaining at the beginning of the interview that the interview is not a clinical consultation; however, any questions can be answered at the end of the interview.

#### Interview Analysis

The interviews will be digitally audio-recorded and transcribed by a professional transcription company. The data will be entered into a software program to facilitate qualitative analysis (NVivo10, QSR International (UK) Ltd, London, UK). All participants’ names will be removed from the transcripts to retain confidentiality. When writing the results, no quotes will be directly attributed to participants. Participants will be given the opportunity to read and approve the transcript. The transcript will be sent to participants, and if no response is heard within 2 weeks, the transcript will be included in the analysis.

The results from the interview will be analyzed using a thematic and framework analysis, which uses a 5-step approach to analyzing and writing up data [11]. This involves familiarization with the interviews, identifying themes, indexing the themes onto the interview transcripts, charting, and mapping the themes. The themes from the patient and health professionals’ interviews can be compared. This will enable key themes to be systematically identified and to map the themes from the patients against those from the health care professionals. A random sample of 5 interviews will also be independently assessed by the supervisor to review the themes and analysis undertaken.

The interviews will take between 45 min to 1 hour to complete depending on what the participants share with the interviewer. The interview can be terminated at any point the participant wishes to stop, and this will not influence their subsequent treatment.

### Sample Size

The study was powered to show a difference between the MISSION group and the primary care group for the primary outcome, exacerbation frequency. The sample size was based on an analysis of covariance (ANCOVA) approach, with the outcome being exacerbation frequency in the second 6-month period and with the exacerbation frequency in the first 6-month period being the covariate. Baseline data suggest a mean number of exacerbations for this group of 1.29, with an SD of 1.30. There is expected to be no change in exacerbation frequency between time periods for the primary care group, whereas it is anticipated that exacerbations in the MISSION group may reduce by 50% to 0.65 exacerbations. A correlation between the exacerbation rates in the 2 time periods of 0.5 is assumed. The primary care group will be larger, with an anticipated 3:1 ratio. To show a difference between groups with a 5% significance level and 90% power, it is calculated that 44 subjects in the MISSION group and 132 in the primary group are required.

### Data Analysis

#### Description of Analysis Populations

All data collected will be analyzed. There will be an analysis of the MISSION and outpatient groups separately. We will analyze 4 groups of participants: (1) qualitative assessments in participants attending the severe clinic, (2) health professionals attending either of the rapid or severe clinics, (3) quantitative assessments between those attending the rapid clinic in primary care to those invited but did not attend, and (4) those who attended the severe clinic compared with those routinely attending the outpatient severe asthma clinics in hospital.

#### Analysis of Endpoints

The analyses will compare the study outcomes between the 4 study groups.

The primary outcome is exacerbation frequency. This outcome will be analyzed using ANCOVA, with the outcome variable being exacerbation frequency in the second 6-month period and the exacerbation rate in the first 6-month period treated as the covariate. Owing to the likely skewed distribution of the exacerbation frequency, it may be necessary to perform a log transformation of the outcome before analysis. Comparisons will be made overall between the 3 groups, and specifically between the MISSION group and each of the other 2 groups.

The analysis of secondary outcomes measured on a categorical scale during the second 6-month period will be compared between groups using Fisher exact test. Continuous variables will be compared between groups using analysis of variance, if found to be normally distributed, or the Kruskal-Wallis test, if not found to be normal.

Comparisons between groups will also be made accounting for the values during the first time period. ANCOVA will be used for continuous variables, whereas logistic regression will be used for binary outcomes.

### Discontinuation and Withdrawal of Participants From Study Treatment

Participants may withdraw at any point in either the qualitative or quantitative aspects of the study.

### Definition of End of Study

The end of study is the date of the last qualitative interview of the last participant.

### Procedure for Dealing with Missing, Unused, and Spurious Data

All data collected will be included in the analysis.

### Procedures for Reporting Any Deviation(s) From the Original Statistical Analysis Plan

The study will analyze observational data retrospectively, and so we do not anticipate any significant deviations as would be expected in a longitudinal study.

### Interim Analysis and Criteria for Early Study Termination

No interim analyses will be performed.

### Dissemination

Patient volunteers and patients who have taken part in the qualitative interviews will be invited to help with dissemination events. The results will be submitted for presentation at national conferences. There will also be reports on the Wessex Asthma Network website for patients and public to access [12]. All study participants will be given a summary of the study results in an appropriate format.

## Results

### Quantitative

The MISSION team screened GP records to identify uncontrolled asthmatics to invite to MISSION clinics. There were 369 patients identified who had one or more of the criteria that suggest uncontrolled or potentially severe asthma (high inhaled corticosteroid dose >500 mcg BDP (Beclomethasone Diproprionate) , 2 or more exacerbations in the last 12 months requiring steroids for more than 3 days, any hospital, ED or OOH contact in the last 12 months, and high SABA usage—>6 inhalers in the last 12 months or reduced lung function FEV1 ≤70%). All patients who were identified were approached for the MISSION project.

### Modern Innovative Solutions to Improve Outcomes in Rapid Access Asthma Clinic Patients and Severe Asthma Assessment Clinic Patients

The MISSION service: The searches identified 1436 patients who were manually reviewed by the MISSION team and 460 patients met the criteria. All these patients were invited to the clinic; 125 were booked in, and 84 attended the RAACs, of whom 22 were identified for the SAACs.

### Primary Care Patients

All patients identified by the GP search but who did not attend the MISSION clinics will be approached for the study to maximize the chance of getting the same number of participants to the number who consents from the MISSION group.

### Outpatient Severe Asthma Patients

The severe asthma outpatient clinic at Portsmouth Hospitals NHS Trust sees 16 new referrals per month. The sample will be matched to the same as the number of patients attending the SAACs, that is, 20 patients. All new referrals to the asthma clinic who meet the inclusion criteria will be included until the sample size is reached.

### Qualitative

All 20 patients from the MISSION SAACs will be approached for a qualitative interview. Furthermore, all clinical staff who attended the MISSION RAACs or SAACs will be approached for an interview with an anticipated number of 10 interviewed from primary care and 10 from secondary care.

## Discussion

The clinical intervention has been performed and the study has been ethically approved by the Yorkshire & The Humber-Leeds East Research Ethics Committee (Reference 15/YH/0136) and sponsored by Portsmouth Hospitals NHS Trust. The results will be reported in February 2020.

## Appendix

Multimedia Appendix 1 The study flow chart showing participants’ journey.

Multimedia Appendix 2 The case record form used for the Modern Innovative Solutions to Improve Outcomes Severe Asthma Assessment Clinics.

## Abbreviations

ACQ: Asthma Control Questionnaire
ANCOVA: analysis of covariance
AQLQ: Asthma Quality of Life Questionnaire
CT: computed tomography
ED: emergency department
ENT: ear, nose, and throat
FeNO: fractional exhaled nitric oxide
FEV1: forced expiratory volume in 1 second
FVC: forced vital capacity
GP: general practitioner
MDT: multidisciplinary team
MISSION: Modern Innovative Solutions to Improve Outcomes
NHS: National Health Service
OOH: out-of-hour
PIS: patient information sheet
RAAC: Rapid Access Asthma Clinic
RCT: randomized controlled trial
SAAC: Severe Asthma Assessment Clinic
SABA: short-acting beta-agonist
