# Supplementary figures and images for: Modern Innovative Solutions to Improve Outcomes in Severe Asthma: Protocol for a Mixed Methods Observational Comparison of Clinical Outcomes in MISSION Versus Current Care Delivery
Source: JMIR Res Protoc. 2019 Oct 10;8(10):e9585. doi: 10.2196/resprot.9585 (PMC6913683; doi:10.2196/resprot.9585)

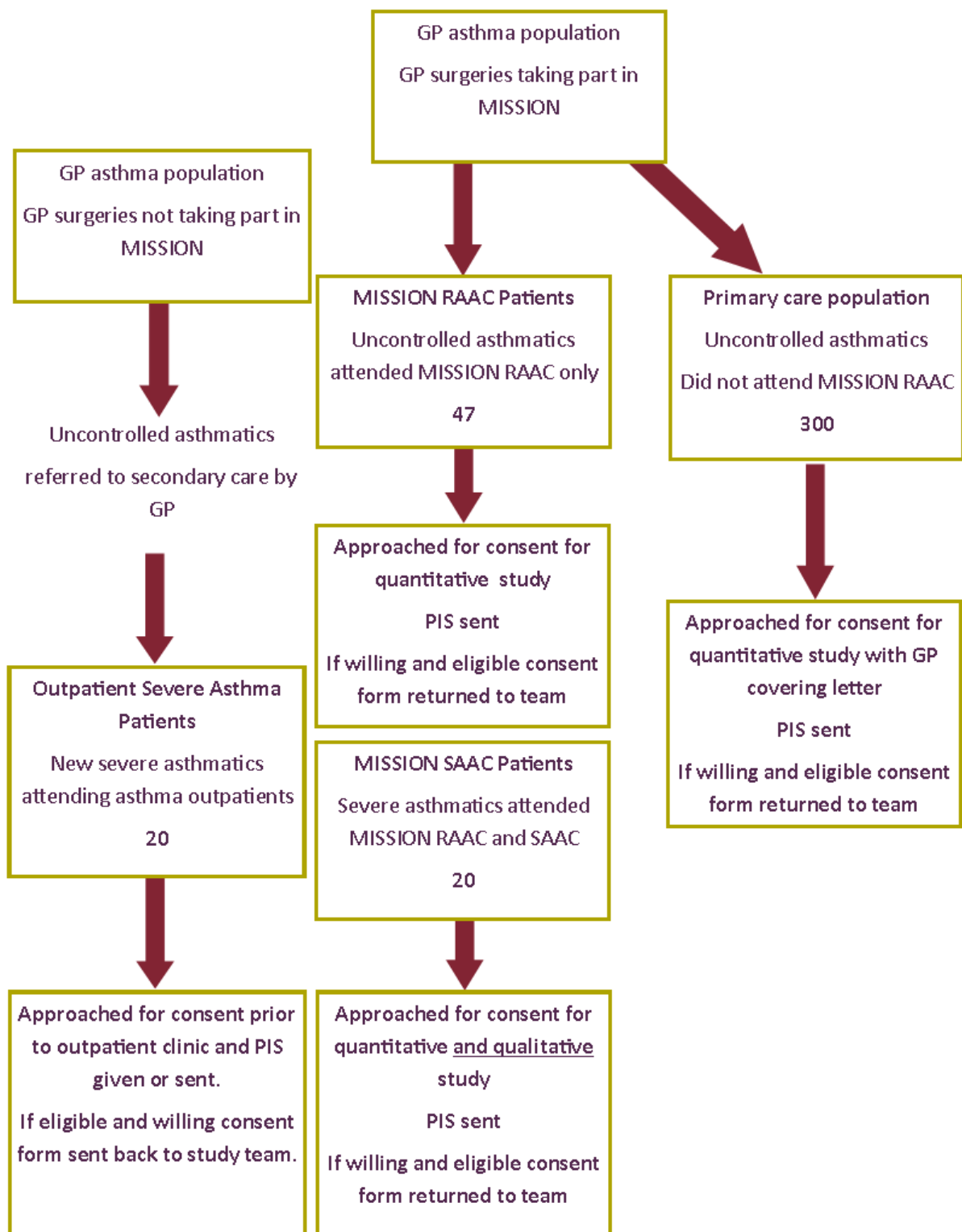

Supplement: Multimedia Appendix 1 [file resprot_v8i10e9585_app1.pdf]
