# Supplementary material for: Modern Innovative Solutions to Improve Outcomes in Severe Asthma: Protocol for a Mixed Methods Observational Comparison of Clinical Outcomes in MISSION Versus Current Care Delivery
Source: JMIR Res Protoc. 2019 Oct 10;8(10):e9585. doi: 10.2196/resprot.9585 (PMC6913683; doi:10.2196/resprot.9585)
Supplement: Multimedia Appendix 2 [file resprot_v8i10e9585_app2.pdf]

Wessex Asthma Network

Portsmouth Hospitals NHS Trust

RAPID ACCESS ASTHMA CLINIC CRF INDEX

Station	Page number	Complete – please sign
Patient data/height/weight	2	
Medical review	3	
Spirometry	11	
Inhaler technique and peak flow	12	
Questionnaires	13	
Skin prick testing	14	
Self management plans	15	
Education	16	
Diagnosis	17	
Treatment	18	
Follow up	19	

WELCOME - PATIENT DATA/HEIGHT/WEIGHT

**1. Patient Name**		
	**Address**	
**2. Patient's Date of Birth:**		DD/MM/YYYY
	**Age**	
**3. Gender**		☐ **Male** ☐ **Female**
**4. Height**		☐ . ☐ ☐ **m**

**5. Weight**				.		**Kg**
**6. BMI**				.		**Kg/m²**

**STATION – MEDICAL REVIEW**	
**1. Asthma history including date of diagnosis**	
Wheeze	☐ **Yes** ☐ **No**
Cough	☐ **Yes** ☐ **No**
Shortness of breath	☐ **Yes** ☐ **No**

**2. Exacerbation history last 12 months**		
Steroid courses – oral or IM	☐ **Yes** ☐ **No** **Number** ______	
Exacerbations managed by GP	☐ **Yes** ☐ **No** **Number** ______	
Exacerbations requiring nebuliser	☐ **Yes** ☐ **No** **Number** ______	
Attendances at ED	☐ **Yes** ☐ **No** **Number** ______	
Hospital admissions	☐ **Yes** ☐ **No** **Number** ______	
HDU/Respiratory High Care admissions	☐ **Yes** ☐ **No** **Number** ______	
ITU admission	☐ **Yes** ☐ **No** **Number** ______	
**Hospital**	**Admission Date**	**Discharge Date**

**3. Allergy triggers**		**Symptom on exposure, please tick**		
		**Eyes***	**Nose***	**Chest***
House dust mite eg vacuuming, dusting		☐ **Yes** ☐ **No**	☐ **Yes** ☐ **No**	☐ **Yes** ☐ **No**
Cat		☐ **Yes** ☐ **No**	☐ **Yes** ☐ **No**	☐ **Yes** ☐ **No**
Cat at home	☐ **Yes** ☐ **No**			
Dog		☐ **Yes** ☐ **No**	☐ **Yes** ☐ **No**	☐ **Yes** ☐ **No**
Dog at home	☐ **Yes** ☐ **No**			
Mould		☐ **Yes** ☐ **No**	☐ **Yes** ☐ **No**	☐ **Yes** ☐ **No**
Grass		☐ **Yes** ☐ **No**	☐ **Yes** ☐ **No**	☐ **Yes** ☐ **No**
Trees		☐ **Yes** ☐ **No**	☐ **Yes** ☐ **No**	☐ **Yes** ☐ **No**
Other		☐ **Yes** ☐ **No**	☐ **Yes** ☐ **No**	☐ **Yes** ☐ **No**
*Eye symptoms – redness,itching, watering<br>*Nasal symptoms – runny, itchy, stuffy nose/sneezing<br>*Chest symptoms – wheeze, cough, chest tightness, breathlessness				

**4. Other atopy**	
Eczema	☐ **Yes** ☐ **No**
Migraine	☐ **Yes** ☐ **No**

**5. Other asthma triggers – *Patient to complete***	
Infections (viral/common cold, etc.)	☐ **Yes** ☐ **No**
Cigarette smoke	☐ **Yes** ☐ **No**
Exercise	☐ **Yes** ☐ **No**
Stress	☐ **Yes** ☐ **No**
Temperature changes	☐ **Yes** ☐ **No**
Drugs (eg NSAIDs/beta blockers)	☐ **Yes** ☐ **No**
Food	☐ **Yes** ☐ **No**
Alcohol	☐ **Yes** ☐ **No**
Hormonal (pre-menstrual/pregnancy)	☐ **Yes** ☐ **No**
Other	☐ **Yes** ☐ **No**
Please specify: ______________________________	

**6. Occupation**			
Retired	☐ **Yes** ☐ **No**	Unemployed	☐ **Yes** ☐ **No**
**7. Any exposure to fumes, chemicals, aerosols or dust at work**			☐ **Yes** ☐ **No**
Details: ______________________________<br>______________________________			
**8. Are asthma symptoms worse at work or improve on holiday**			☐ **Yes** ☐ **No** ☐ **N/A**
Details: ______________________________<br>______________________________			

**9. Any hobbies that may cause exposure to allergic triggers, chemicals, dust, mould** (eg horse riding, soldering, woodwind instruments, woodwork, DIY)	☐ **Yes** ☐ **No**
Details:	
**10. Are asthma symptoms triggered by hobbies**	☐ **Yes** ☐ **No** ☐ **N/A**
Details:	

**11. Past medical history**	
**Cardiovascular disease**	☐ **Yes** ☐ **No**
Please specify:	
**Other respiratory disease**	☐ **Yes** ☐ **No**
Please specify:	
**ENT disease**	☐ **Yes** ☐ **No**
Please specify:	
**GI/abdominal disease**	☐ **Yes** ☐ **No**
Please specify:	
**Other**	☐ **Yes** ☐ **No**

Please specify:

**12. Drug allergy**	☐ **Yes** ☐ **No**
**Details**	
**13. History of anaphylaxis**	☐ **Yes** ☐ **No**
**Details**	

14. Current medications, inhalers, nebulisers, nasal sprays and topical medication including over the counter medication

**Drug**<br>*Generic name in CAPITALS*	**Prescribed dose**<br>*Include units and frequency*	**OTC or GP**	**Route**<br>*Po/sc/pr/ patch/neb/ inh*	**With spacer**<br>*Y/N/NA*	**Adherent**<br>*Y/N*

**15. Smoking status**			
**Current smoker**	☐	**Pack years**	___________
**Ex-smoker**	☐	Pack year defined as 20 cigarettes per day for 1 year	
**Never smoked**	☐		

**16. If smoker, expired CO**				**ppb**

STATION - SPIROMETRY

**1. Lung function**	**FEV1**	**Litres**
Attach spirometry chart below	**FEV1 % predicted**	**%**
	**FVC**	**Litres**
	**FVC % predicted**	**%**
	**FEV1/FVC ratio**	**%**
	**Peak flow**	**l/min**
	**Predicted peak flow**	**l/min**

**2. FeNO**				**ppb**

STATION – INHALER TECHNIQUE AND PEAK FLOW

**1. Has patient brought their inhalers in**		☐ **Yes** ☐ **No**
**2. Inspiratory flow checked**		☐ **Yes** ☐ **No**
**3. Current inhaler devices**	MDI alone	☐ L/Min
	MDI plus spacer	☐ L/Min

	Turbohaler	☐	L/Min
	Accuhaler	☐	L/Min
	Other please specify	☐	L/Min
**4. Recommended inhaler device**	MDI alone	☐	
	MDI plus spacer	☐	
	Turbohaler	☐	
	Accuhaler	☐	
	Other please specify	☐	
**5. Inhaler technique checked**		☐ **Yes**	☐ **No**
**6. Inhaler technique improved**		☐ **Yes**	☐ **No**
**7. Inhaler information sheet given**		☐ **Yes**	☐ **No**
**8. Peak flow (mini Wright) – add value to SMP on page 15**			L/Min
**9. Peak flow meter given**		☐ **Yes**	☐ **No**

**STATION - Patient Questionnaires**		
**1. ACQ 7 complete**		☐ **Yes** ☐ **No**
NB. include item 7 FEV1 %predicted	Score:	
**2. AQLQ complete**		☐ **Yes** ☐ **No**
	Score:	
**3. SNOT 22 complete**		☐ **Yes** ☐ **No**
	Score:	

4. **Epworth complete**	☐ **Yes** ☐ **No**
	Score:
5. **HADS complete**	☐ **Yes** ☐ **No**
	Score: **Anxiety** ☐ **Depression** ☐
6. **Nijmegen complete**	☐ **Yes** ☐ **No**
	Score:
7. **GERDQ complete**	☐ **Yes** ☐ **No**
	Score:
8. **Patient survey complete**	☐ **Yes** ☐ **No**
	Score:

STATION – Skin Prick Testing and SMP

**1. ANY HISTORY OF ANAPHYLAXIS? *IF YES, AVOID SKIN PRICK TEST***	☐ **Yes** ☐ **No**
**2. On antihistamines? *If yes, interpret with caution***	☐ **Yes** ☐ **No**

**3. Type of test**	Result (please circle)		Wheal (size in mm)
**Positive control**	positive	negative	X mm
**Negative control**	positive	negative	X mm
**Cat**	positive	negative	X mm
**Dog**	positive	negative	X mm
**House dust mite**	positive	negative	X mm
**Aspergillus**	positive	negative	X mm

**Grass pollen**	positive	negative	X mm
**Tree pollen**	positive	negative	X mm

**QUESTIONAIRRES**	
**1. Remind patient to complete questionnaires**	☐ **Yes** ☐ **No**

**SMP**	
**1. Self-management plan devised**	☐ **Yes** ☐ **No**
**2. Peak flow on mini Wright (see page 12)**	
**3. Predicated peak flow**	
**4. Peak flow 75 – 85%**	
**5. Peak flow 50 – 75%**	
**6. Peak flow <50%**	

**STATION – Education session**	
**1. Patient attended**	☐ **Yes** ☐ **No**
**2. Partner or Carer(s) attended**	☐ **Yes** ☐ **No**

**Education content**	
**1. Asthma education and triggers**	☐ **Yes** ☐ **No**
**2. Asthma self-management**	☐ **Yes** ☐ **No**
**3. Smoking cessation**	☐ **Yes** ☐ **No**

SMP	
**Self-management plan devised**	☐ Yes ☐ No

OUTCOME - Diagnosis		
**1. Asthma?**		☐ Yes ☐ No
If no, please specify		
**2. Controlled?**		☐ Yes ☐ No
**3. Allergic?**		☐ Yes ☐ No
**4. Inflammation?**		☐ Yes ☐ No
**5. Co - morbidity?**		☐ Yes ☐ No
	**GORD?**	☐ Yes ☐ No
	**Rhinosinusitis?**	☐ Yes ☐ No
	**Other?**	☐ Yes ☐ No
Details		

OUTCOME - Treatment	
**1. Any recommended change to inhaled medication?**	☐ Yes ☐ No
	☐ Step up ☐ Step down
If yes, please specify:	
**2. Any recommended change to medication adherence?**	☐ Yes ☐ No
If yes, please specify:	

**3. Any recommended change to other medications?**		☐ Yes ☐ No
	**PPI**	☐ Yes ☐ No
	**Nasal corticosteroid**	☐ Yes ☐ No
	**Antihistamine**	☐ Yes ☐ No
	**Other**	☐ Yes ☐ No
Details:		

**OUTCOME – Follow up**	
**1. Further investigations recommended?**	☐ Yes ☐ No
Details:	
**2. Follow up recommendations**	
**GP follow up** *(with 1-year plan)*	☐ Yes ☐ No
**Research clinic follow up**	☐ Yes ☐ No
**SAAC follow up**	☐ Yes ☐ No
**Referral to other secondary care** *(GP to refer)*	☐ Yes ☐ No
Details:	

SECTION 1 FeNO and exhaled CO	
FeNO	ppb
CO	ppb

SECTION 2 - QUESTIONAIRRES

1. ACQ complete		☐ Yes ☐ No
	Score:	
2. AQLQ complete		☐ Yes ☐ No
	Score:	
3. SNOT 22 complete (OPTIONAL)		☐ Yes ☐ No
	Score:	
4. Epworth complete (OPTIONAL)		☐ Yes ☐ No
	Score:	
5. HADS complete (OPTIONAL)		☐ Yes ☐ No
	Score: **Anxiety** ☐	**Depression** ☐
6. Nijmegen complete (OPTIONAL)		☐ Yes ☐ No
	Score:	
7. GERDQ complete (OPTIONAL)		☐ Yes ☐ No
	Score:	

SECTION 3 - ASTHMA HISTORY	
**1.Review of asthma history**	Triggers<br>Exacerbations<br>Medication<br>Occupation<br>Hobbies<br>Co morbidity<br>Reflux<br>Past medical history<br>smoking

**2. Relevant past medical history**

**3. Drug allergy**	☐ **Yes** ☐ **No**
**Details**	
**4. History of anaphylaxis**	☐ **Yes** ☐ **No**
**Details**	

5. Current medications, inhalers, nebulisers, nasal sprays and topical medication including over the counter medication

**Drug**<br>*Generic name in CAPITALS*	**Prescribed dose**<br>*Include units and frequency*	**OTC or GP**	**Route**<br>*Po/sc/pr/ patch/neb/ inh*	**With spacer**<br>*Y/N/NA*	**Adherent**<br>*Y/N*

SECTION 4 - RADIOLOGY	
**1. Chest x-ray**	☐ **Yes** ☐ **No**
**Result:**	
**2. HRCT**	☐ **Yes** ☐ **No**
**Result:**	
**3. CT sinus**	☐ **Yes** ☐ **No**

Result:

SECTION 5 - PATHOLOGY		
**1. Blood tests taken today**		☐ Yes ☐ No
Please specify:		
**Test**	**Date**	**Result**
WCC		
Eosinophils		
Total IgE		
Aspergillus IgE		
Aspergillus IgG		

**2. Skin Prick test**			
**Skin prick test done at RAAC**			☐ Yes ☐ No
**Result:**			
If no or inconclusive then repeat or send venous sample			
**3. ANY HISTORY OF ANAPHYLAXIS?** ***IF YES, AVOID SKIN PRICK TEST***			☐ Yes ☐ No
**4. On antihistamines?** ***If yes, interpret with caution***			☐ Yes ☐ No
**5. Type of test**	Result (please circle)		Wheal (size in mm)
**Positive control**	positive	negative	X mm

**Negative control**	positive	negative	X mm
**Cat**	positive	negative	X mm
**Dog**	positive	negative	X mm
**House dust mite**	positive	negative	X mm
**Aspergillus**	positive	negative	X mm
**Grass pollen**	positive	negative	X mm
**Tree pollen**	positive	negative	X mm
	positive	negative	X mm
	positive	negative	X mm
	positive	negative	X mm
	positive	negative	X mm
	positive	negative	X mm
	positive	negative	X mm

SECTION 6 - LUNG FUNCTION	
**1. Spirometry**	☐ **Yes** ☐ **No**
**2. Lung volume**	☐ **Yes** ☐ **No**
**3. Resistance**	☐ **Yes** ☐ **No**
**4. Reversibility**	☐ **Yes** ☐ **No**
Please file lung function report in notes	

SECTION 7 - INHALER TECHNIQUE AND SMOKING CESSATION	
**1. Has patient brought their inhalers in**	☐ **Yes** ☐ **No**
**2. Inspiratory flow checked**	☐ **Yes** ☐ **No**

**3. Current inhaler devices**	MDI alone	☐	L/Min
	MDI plus spacer	☐	L/Min
	Turbohaler	☐	L/Min
	Accuhaler	☐	L/Min
	Other please specify	☐	L/Min
**4. Recommended inhaler device**	MDI alone	☐	L/Min
	MDI plus spacer	☐	L/Min
	Turbohaler	☐	L/Min
	Accuhaler	☐	L/Min
	Other please specify	☐	L/Min
**5. Inhaler technique checked**		☐ **Yes**	☐ **No**
**6. Inhaler technique improved**		☐ **Yes**	☐ **No**
**7. Inhaler information sheet given**		☐ **Yes**	☐ **No**

**8. Smoking cessation advice given**	☐ **Yes** ☐ **No** ☐ **N/A**
**Details:**	

SECTION 8 - DIETICIAN

**BMI**			**.**		**Kg/m²**		**Triggers**<br>**Salicylates**<br>**Sulphites**<br>**Reflux**

	Oral allergy<br>Weight

SECTION 9 - PHYSIOTHERAPY	
Nijmegen /64	

SECTION 10 - PSYCHOLOGY		
HADS Score:	**Anxiety**	**Depression**

SECTION 11 - ENT		
**SNOT 22 score**	**Nasendoscopy**	☐ **Yes** ☐ **No**

SECTION 12 – EXIT MEDICAL REVIEW

SECTION 13 - MDT
**Case summary- Medical**
**Lung function**
**Inhaler technique**
**Dietician**
**Physiotherapy**

**Psychology**
**ENT**
**Co morbidities**

**a.** Rhinitis	☐ **Yes**	☐ **No**	☐ **Possible**
**b.** Food Allergy	☐ **Yes**	☐ **No**	☐ **Possible**
**c.** Anxiety/Depression	☐ **Yes**	☐ **No**	☐ **Possible**
**d.** GORD	☐ **Yes**	☐ **No**	☐ **Possible**
**e.** Hyperventilation	☐ **Yes**	☐ **No**	☐ **Possible**
**f.** Obesity	☐ **Yes**	☐ **No**	
**g.** VCD	☐ **Yes**	☐ **No**	☐ **Possible**
**h.** ABPA	☐ **Yes**	☐ **No**	☐ **Possible**
**i.** Asthma/COPD overlap syndrome	☐ **Yes**	☐ **No**	☐ **Possible**
**j.** NSAID sensitivity	☐ **Yes**	☐ **No**	☐ **Possible**
**k.** Eczema	☐ **Yes**	☐ **No**	☐ **Possible**

Any other significant co-morbidities likely to affect asthma:

____

____

____

MDT RECOMMENDATIONS
**Medication**
**Investigations**
**Follow up**
**Research**
